# Superior Canal Dehiscence Syndrome

**DOI:** 10.1016/S1808-8694(15)30978-2

**Published:** 2015-10-19

**Authors:** Suzane da Cunha Ferreira, Marco Antonio de Melo Tavares de Lima

**Affiliations:** a MD, 2nd year otolaryngology resident at Hospital da Lagoa-RJ; b PhD in Medicine, Associate Professor at the Department of Otorhinolaryngology and Ophthalmology of the Federal University of Rio de Janeiro. Otolaryngologist at the Hospital da Lagoa - Rio de Janeiro

**Keywords:** superior canal dehiscence, conductive hearing loss, dizziness, vertigo

## Abstract

The Superior Canal Dehiscence Syndrome (SCDS) was first reported by Minor at. Al. (1998), and has been characterized by vertigo and vertical-torsional eye movements related to loud sounds or stimuli that change middle ear or intracranial pressure. Hearing loss, for the most part with conductive patterns on audiometry, may be present in this syndrome. We performed a literature survey in order to to present symptoms, signs, diagnostic and therapeutic approaches to the SCDS, also aiming at stressing the great importance of including this syndrome among the tractable cause of vertigo. We should emphasize that this is a recent issue, still unknown by some specialists. The Correct SCDS diagnosis, besides enabling patient treatment, precludes misdiagnosis and inadequate therapeutic approaches.

## INTRODUCTION

The Superior Semicircular Canal Dehiscence Syndrome (SSCDS) is a rare disease, mainly characterized by vestibular symptoms induced by intense sound stimuli or by changes in intracranial or middle ear pressure, due to a dehiscence of the bony layer that covers the superior semicircular canal. Although less frequent, some individuals with SSCDS only have hearing loss, and no vestibular symptoms.

Since this is a recently described syndrome, most heath care professionals who work with these patients are still not used to its diagnosis and it should be part of vertigo differential diagnosis, and even of isolated hearing loss differential diagnosis.

This paper aims at educating these professionals on the importance of this syndrome, discussing its main clinical, diagnostic and therapeutic issues through a literature review on the subject.

## LITERATURE REVIEW

The Superior Semicircular Canal Dehiscence Syndrome (SSCDS) was first described in 1998 by Minor et al. Aiming at showing the symptoms, nystagmus patterns and temporal bone CT Scan results in patients who suffered from vertigo induced by intense sound stimuli or pressure changes in the middle ear (ME) or intracranialy (IC). They carried out a prospective study with a series of cases and found 8 patients who presented ocular movements in a plane parallel to the superior semicircular canal, evoked by pressure and sound stimuli, as described above. Such findings coincided with the superior semicircular canal dehiscence images found on high resolution temporal bone CT scan of these 8 individuals^1^.

After that, Smullen, Andrist and Gianoli published 3 cases of patients with temporal bone CT scan results compatible with superior Semicircular canal dehiscence syndrome, with later surgical confirmation of such alteration. Two of these patients suffered from incapacitating vertigo and improved after surgical repair of the dehiscence through middle fossa access^2^.

Aiming at correlating signs, symptoms and findings of diagnostic tests, and describe the surgical procedure used to repair the dehiscence, Minor selected other 17 individuals with clinical and radiological signs of SSCDS. Five of these patients presented incapacitating vestibular symptoms and underwent neurosurgery via middle fossa to repair the lesion. Three of them suffered total obliteration of the superior semicircular canal (plugging). The other two needed bone and fascia grafts over the dehiscence site, keeping the canal permeable. All of them experienced improvements in their symptoms, although one of them developed sensorineural hearing loss in the immediate postoperative, and another one developed vestibular hypofunction in the operated side. None of the two techniques employed seemed better than the other in this study. An interesting finding from this author was that ten of the seventeen patients reported a triggering event for the symptoms (4 by head injury and 6 by sudden pressure changes in the middle ear or intracranialy)^3^.

Carey, Minor and Nager studied 1000 temporal bones (596 individuals) obtained from autopsies in a randomized fashion, under the microscope, in order to determine SSCDS prevalence in the general population. 0.7% of the individuals had complete dehiscence of the superior canal and 1.3% had a very thin bony layer (0.1mm or less) covering the canal. In most of the specimens the findings were bilateral. When they analyzed the temporal bones of children, the same authors concluded that they usually presented a thinning of the bone layer over the superior semicircular canal, and the adult thickness is reached at about 3 years of age. They then postulated that a defect on this bony layer development would be responsible for the thin layer of bone found in some adult individuals. An injury of some kind could then rupture this thin bony layer, thus causing the dehiscence itself^4^.

Cremer et al. made a 3D study through magnetic search coils, of the ocular movements evoked by sound stimuli and Valsalva maneuver in 11 individuals with SSCDS. After analyzing the responses obtained, they concluded that these movements truly came from the superior semicircular canal, and this reinforced the causal relationship between signs, symptoms and the anatomic alteration^5^.

Brantberg et al. presented other 8 patients with SSCDS signs and symptoms confirmed by high resolution CT scan. Two of them underwent transmastoid dehiscence repair surgery through superior semicircular canal plugging and had significant symptoms improvement^6^.

Minor et al. published a series of 28 cases of patients with SSCDS clinical and radiological signs and symptoms, gathered during a 6 year study at a tertiary institution. At the same time, Hirvonen et al. investigated triggering frequency changes in the vestibular nerve in response to changes in the external acoustic meatus (EAM) pressure, before and after superior canal fenestration in chinchillas. Before fenestration, only one of the 9 superior canals studied responded to pressure changes. After fenestration all of them could be inhibited by negative pressure and excited by positive pressure on the EAM. Such responses were abolished when the windows were occluded with sealing material. The results led to the conclusion that a fenestration on the superior semicircular canals makes them more sensitive to pressure. Moreover, response obtained from the pressure stimuli over the EAM coincide with the endolymph flow model known for the superior canal^7^, ^8^.

Belden et al., comparing the prevalence of superior canal dehiscence shown on temporal bone CT scan of 50 symptomatic patients with 107 in the control group using 0.5mm and 1.0mm slices state that the positive predictive value of this exam to identify SSCDS is better with spiral CT scan, with 0.5mm slices and reconstruction at the plane of the superior semicircular canal. Williamson et al., aiming at also defining SSCDS prevalence reached similar conclusions after retrospectively analyzing 442 temporal bone CT Scans of patients with the most varied symptoms. SSCDS prevalence found using 1.0mm coronal cross-sections was substantially higher than the prevalence found in the temporal bone histology studies. Moreover, most of the individuals considered with SSCDS on the CT scan of this study did not present clinical symptoms of the disease. Therefore, the number of false positives could increase exponentially when 1.0mm temporal bone CT scan slices are used, and when image results are not correlated to clinical signs and symptoms^9^, ^10^.

Minor et al. published 4 cases of patients with superior semicircular canal syndrome seen on the temporal bone CT scan who had air-bone audiometric gap. Three of them had been previously submitted to stapedectomy, without hearing improvements. The fourth underwent exploratory tympanotomy, without evidence of middle ear alterations. They concluded that SSCDS may be the cause of the apparently conductive hearing loss. A mobile “third window” created by the dehiscence would be responsible for acoustic energy dissipation, thus causing the conductive hearing loss^11^.

Krombach et al. studied the temporal bone CT scans of 507 patients and showed that the posterior semicircular canal dehiscence has a similar prevalence to that of the superior canal. Aiming at determining the prevalence of the posterior semicircular canal dehiscence with clinical symptoms, they analyzed the CT scans of 128 patients with vertigo, 183 patients with sensorineural hearing loss and 196 individuals without inner ear symptoms. They also concluded that the dehiscence findings were much more common in individuals with previous history of vertigo when compared to asymptomatic persons^12^.

In a recent study, Mikulec et al., aiming at describing the superior semicircular canal dehiscence as a conductive hearing loss cause, gathered other 8 cases (total of 10 ears) with SSCDS shown at the temporal bone CT scans and with audiometric profiles matching those with conductive hearing loss coinciding with the dehiscence side. None of them reported vestibular symptoms. The air-bone gap was more significant in the lower frequencies. Acoustic reflexes and the vestibular evoked myogenic potential (VEMP) were present, though expected to be absent, in the case of conductive hearing loss caused by middle ear alterations. Exploratory tympanotomy had been carried out in six ears and were negative for alteration findings. Of these six, three of them had undergone stapes surgery, without hearing improvement. Therefore, the superior semicircular canal dehiscence may present itself as conductive hearing loss and simulate otosclerosis, for instance. Since two of these subjects were siblings, it was thought that genetic factors may be involved in the genesis of this alteration in the inner ear^13^.

## DISCUSSION

The prevalence of complete dehiscence of the superior semicircular canal is estimated to be of 0.7% in the general population, while 1.3% has a bony layer of less than 0.1mm covering the canal (and this would be considered dehiscent at high resolution temporal bone CT scans by current standards). It is important to remember that not all patients with SSCDS have the syndrome symptoms, and we still do not know the percentage of symptomatic patients among them^4^.

Gender did not prove to be of statistical significance in none of the studies, and the age median of symptomatic patients was around 40 years for Minor and 41 for Minor et al. There was also no report of children being affected. There are cases described of siblings being affected, and this may lead us to consider some genetic factor associated to SSCDS development^3^, ^7^, ^13^.

The anatomical alteration etiopathology is still unknown, although it has been postulated that the defect could occur during the development of the bony layer that covers the semicircular canal (until 3 years of age). A second event, for instance a head injury or a sudden increase in intracranial pressure, could cause the rupture of this abnormally thin layer and complete the dehiscence^3^, ^4^, ^14^.

Most of these individuals sought medical help because of vestibular symptoms such as chronic unbalance and vertigo. Although SSCDS bears a close relationship with vertigo or oscilopsia and the exposure to high intensity sound stimuli, most patients did not report symptoms worsening with noise if they were not questioned about it. The same symptoms happened to many of them when they made movements that changed the pressure gradient within the middle ear or intracranialy (blow one's nose, cough, raise heavy objects, press on the tragus, etc). Moreover, some of them reported they could hear in the affected side, the movements of their own eyes, their heart beats and cracks when they moved their joints. Very few of them went to see an otorhinolaryngologist having only hearing loss, without vestibular symptoms^1^, ^3^, ^5^, ^7^, ^13^, ^15^, ^16^.

Most of the patients presented rotational and vertical nystagmus evoked by intense sound stimuli (100 to 110 dB NA in frequencies that varied between 250 and 4000 Hz) or after maneuvers that would change middle ear or intracranial pressure. It has been postulated that the canal bony dehiscence would work as a “third window” within the inner ear. The mobility of this third window would allow the deflection of the superior canal cupula. The direction of the vertical and rotational nystagmus components depend on the effect of these stimuli over the superior semicircular canal ampulla. Thus, positive pressure within the external acoustic meatus, Valsalva maneuver against nasal constriction and high intensity sounds would cause an ampullifugal movement (excitatory) of endolymph. The resulting nystagmus would be vertical and rotational, with the slow component pointing upwards and towards the opposite side of the affected ear. The ocular movements would have an opposite direction if the stimulus caused ampullipetal movement of the endolymph (inhibitory), which occurs with negative pressure in the external acoustic meatus, Valsalva maneuver against the closed glottis or Jugular vein compression (causing an increase in intracranial pressure)^1^, ^3^, ^5^, ^17^, ^21^.

Physical exam is of fundamental importance, when tests considered standard for the vestibular apparatus investigation such as electronystagmography and rotatory chair failed to show significant alterations. Frenzel goggles should be used in these patients to search for nystagmus in order to suppress the ocular fixation effect^1^, ^3^, ^5^, ^7^.

Tonal audiometry shows “conductive” hearing loss with air-bone gap of 5-10 dB in two or more frequencies (specially on the lower tones) as the most frequently found alteration in symptomatic patients. Some presented even greater air-bone gaps and were misclassified as patients with otosclerosis. Notwithstanding, they had preserved acoustic reflexes in their tympanometries and responded to the vestibular evoked myogenic potential (VEMP), and this made such diagnosis questionable. Mild to moderate sensorineural hearing loss and conductive hearing loss (reduction in the bone conduction threshold, however with air conduction within normal limits) were less frequently observed. We imagine that the acoustic energy dissipation through the canal dehiscence could be responsible for the apparent conductive hearing deficit^1^, ^3^, ^11^, ^13^, ^16^, ^22^, ^23^.

The diagnosis of SSCDS is confirmed through high resolution temporal bone CT scan. In order to reduce the number of false-positives, it is advisable to use 0.5mm slices and image reconstruction at the superior semicircular canal plane. MRI may also be used; however it bears 96% sensitivity and 98% specificity in relation to CT scan^3^, ^9^, ^10^, ^15^, ^24^.

Patients with SSCDS who suffered with incapacitating vestibular symptoms underwent a surgical procedure to repair the dehiscence. Access was made through the middle cerebral fossa in most cases, and together with the neurosurgery team. The two most used techniques were total plugging of the superior canal and reconstruction of the bony layer over the canal with fibrin glue, and later plugging with cortical bone. In order to repair the dehiscence over the superior canal (resurfacing), temporal fascia was used over the membranous portion of the canal (without obstructing the lumen); this fascia was then covered with cortical bone and the middle cranial fossa floor was covered by a large piece of temporal fascia. All operated patients had at least an improvement in their incapacitating symptoms, although some of them developed complications such as sensorineural hearing loss or labyrinth hypofunction on the affected side. None of the two techniques seemed superior so far, and we need further studies and more follow up time with the operated patients to determine the long term effect of both procedures^1^, ^3^, ^19^, ^25^, ^27^.

## FINAL COMMENTS

Although it is a rare alteration and there are many still unclear physiopathological aspects, the Superior Semicircular Canal Dehiscence Syndrome must be included among causes of vertigo. It should also be part of the differential diagnosis list of conductive hearing loss, specially in cases of preserved acoustic reflexes.

It is considered a treatable cause of vertigo. Its clinical suspicion bears, at least, the advantage of causing the avoidance of inadequate diagnostic or therapeutic measures (such as the use of anti-vertigo drugs, sacculotomies, exploratory tympanotomies, stapedectomies, etc), usually not indicated in cases of SSCDS.

Signs and symptoms characteristics of SSCDS are rarely obvious at the time of patient admission. Therefore, health care professionals who see these patients should be apt to proceed with the minimum investigative approach in order to make its diagnosis more probable.
